# Consumption of a Probiotic Blend with Vitamin D Improves Immunity, Redox, and Inflammatory State, Decreasing the Rate of Aging—A Pilot Study

**DOI:** 10.3390/biom14111360

**Published:** 2024-10-25

**Authors:** Judith Félix, Adriana Baca, Luz Taboada, Guillermo Álvarez-Calatayud, Mónica De la Fuente

**Affiliations:** 1 Department of Genetics, Physiology and Microbiology (Animal Physiology Unit), Faculty of Biological Sciences, Complutense University of Madrid, José Antonio Novais, 12, 28040 Madrid, Spain; adriabac@ucm.es (A.B.); mondelaf@ucm.es (M.D.l.F.); 2 Institute of Investigation Hospital 12 Octubre (imas12), 28041 Madrid, Spain; 3 General Medicine Area, Hospital HM Sanchinarro, 28040 Madrid, Spain; luz_taboada@yahoo.es; 4 Gastroenterology and Child Nutrition Area, General University Hospital Gregorio Marañón, 28007 Madrid, Spain; galvarezcalatayud@gmail.com; 5 Department of Medicine, Faculty of Biomedical and Health Sciences, Universidad Europea de Madrid, 28040 Madrid, Spain

**Keywords:** probiotics, biological age, immune system, redox state, inflammatory state, nutritional supplement

## Abstract

There is evidence of the effect of probiotic intake on the immune system. However, the effect probiotics may have on the rate of aging is unknown. The aim of this study is to determine the effect of a probiotic blend on immunity, redox state, inflammation, and the rate of aging or biological age. A group of 10 men and 14 women took, daily for 2 months, a sachet with three probiotics (*Bifidobacterium animalis* subsp. lactis BSO1, *Lactobacillus reuteri* LRE02, *Lactobacillus plantarum* LP14) and vitamin D. Before starting the treatment and after 2 months, peripheral blood was collected. Immune functions were assessed in isolated immune cells, and cytokine concentrations were also measured both in mononuclear cell cultures and plasma. Redox state parameters were also analyzed in whole blood cells. Finally, the Immunity Clock was applied to determine the biological age. Results show that the intake of this probiotic blend in general, in both men and women, improves immunity and decreases the oxidative and inflammatory state. In addition, it rejuvenates the biological age by 10 years on average. It can be concluded that this probiotic blend could be proposed as a good strategy to slow down the aging process, and to achieve healthy aging.

## 1. Introduction

Nowadays, the important role played by the intestinal microbiota in maintaining the health of individuals is becoming increasingly evident [1]. This is because this microbiota not only acts at the intestinal level as a protective barrier against pathogens or carries out metabolic functions but is also known to be closely connected with practically all the organs of the body, contributing in some way to their correct functioning [1,2,3,4,5,6]. However, one of the most important dialogues in which the intestinal microbiota intervenes is with the homeostatic systems (nervous, immune, and endocrine), which are responsible for the maintenance of health [1,7]. Therefore, an alteration of the microbiota, i.e., dysbiosis, could lead to multiple diseases at any level of the organism, such as an increase in infections, the appearance of inflammatory diseases, stress and anxiety disorders, heart, kidney, and skin diseases, etc. [1,2,3,4,5,6].

These alterations of the microbiota can appear due to multiple factors, such as an inadequate diet, stressful situations, after taking antibiotics, obesity, etc. [1,8,9,10,11,12,13,14]. Moreover, this dysbiosis also appears during the aging process (predominant microorganisms generating inflammation and oxidation), accompanied by a deterioration in the functionality of the homeostatic systems, which together generate a chronic oxidative and inflammatory state, thus increasing the morbidity and mortality of the individual [1,7,9,10,11,15]. This aging process is heterogeneous, indicating that each individual is aging at a different rate, that is, they have a different biological age despite possibly having the same chronological age [16]. Based on this, the immune system has been proposed as the best marker of health and as a good indicator of biological age [17]. Indeed, models for predicting biological age have been developed using immune parameters, such as the Immunity Clock [17,18]. Therefore, given the existing communication between microbiota and the immune system, maintaining an adequate microbiota would allow for regulating the correct functioning of the immune system, thus reducing the immunosenescence that occurs with aging, ultimately slowing down the rate of aging [19,20].

However, considering that the consumption of probiotics allows restoration of the altered microbiota, it is understandable that they are increasingly used in the daily life of people to maintain health [21,22]. Thus, it has been observed that the intake of probiotics reduces certain symptoms, not only intestinal, and there is increasing evidence of the beneficial effect that probiotics have on the immune system [22,23,24,25,26,27]. In fact, the term immunobiotic was coined to define those probiotic strains that have a positive effect on the functionality of the immune system [28]. In addition, some strains have also been observed to contribute to healthy aging, even increasing life expectancy in experimental animals. These probiotics that contribute positively to the aging process are known as gerobiotics [29], and their positive effects seem to be associated with their antioxidant and anti-inflammatory properties [30,31].

However, despite the many existing studies on the effects that probiotics can have on the immune system, there are few studies on how probiotics can modulate the rate of aging. Therefore, the aim of the present study was to evaluate the effects of a two-month daily intake of a probiotic blend (*Bifidobacterium animalis* subsp. *lactis* BSO1, *Lactobacillus reuteri* LRE02, *Lactobacillus plantarum* LP14) on immune function, redox state, inflammatory profile, and biological age.

## 2. Materials and Methods

### 2.1. Participants and Extraction of Blood Samples

The participants in this pilot study were initially 13 men and 14 women between 30 and 60 years of age. However, 3 men were excluded from the study due to low adherence to treatment. Therefore, the final experimental N was 10 men and 14 women.

The participants were healthy individuals, i.e., with the absence of pathology or findings of clinical significance in general laboratory parameters. Exclusion criteria were severe general pathology (autoimmune diseases, cancer, anemia, severe allergies, dementia or cognitive impairment, chronic respiratory disease, hypertension, and diabetes), excessive alcohol or drug consumption, hormone replacement therapy, intake of vitamins, antioxidants or any pharmaceutical drug that influences the immune system, as well as low adherence to treatment. In addition, all participants signed an informed consent for the use of their blood samples for research.

The participants took one sachet per day, for 2 months (April–June), of a probiotic supplement containing 3 strains of probiotics (microencapsulated): *Bifidobacterium animalis* subsp. *lactis* BSO1 (LMG P-21384) 2 × 10^9^/sachet; *Lactobacillus reuteri* LRE02 (DSM23878) 0.5 × 10^9^/sachet; *Lactobacillus plantarum* LP14 (DSM33401) 0.5 × 10^9^/sachet and cholecalciferol (Vitamin D3) 1 µg (40 IU), patented without allergens.

Before starting the probiotic supplement and after 2 months of treatment, a survey on well-being was conducted, and blood samples were collected according to the Declaration of Helsinki. Blood samples (12 mL of peripheral blood drawn by vein puncture) were collected from 9:00 am to 10:00 am (in tubes with citrate; BD Vacutainer Systems) to avoid the effect of circadian variations on immune parameters.

This study was approved by the Ethical Committee of the Hospital Clínico San Carlos of Madrid (P.C. P21110b) on 1 March 2022.

### 2.2. Analysis of Immune Function Parameters

#### 2.2.1. Isolation of Neutrophils and Lymphocytes

For the analysis of the chemotactic ability of neutrophils and lymphocytes, neutrophil phagocytosis, natural killer activity, and the lymphoproliferative response in basal and stimulated conditions, neutrophils and lymphocytes were isolated from blood samples following a previously described method [16]. For this, 1.119 and 1.077 density Hystopaque (Sigma-Aldrich, St. Louis, MO, USA) were used for neutrophil and lymphocyte separation, respectively. Collected cells (95% viability determined using trypan blue staining) were adjusted to 10^6^ neutrophils or lymphocytes per mL of Hank’s solution or RPMI 1640 medium (Sigma-Aldrich, St. Louis, MO, USA).

#### 2.2.2. Chemotaxis

The chemotactic ability of neutrophils and lymphocytes was determined according to the Boyden method, with modifications introduced by our group [16]. It is based on the ability of immune cells to migrate to an infectious focus. The cell suspensions were placed into the upper compartment of a Boyden chamber, and f-met-leu-phe (Sigma-Aldrich, St. Louis, MO, USA) was placed in the lower compartment. After a 3 h incubation, the filters were fixed and stained with Giemsa (GIEMSA, PANREAC, Barcelona, Spain). Finally, the chemotaxis index (C.I.) was determined by counting the total number of neutrophils or lymphocytes by optical microscopy (immersion objective) on one-third of the lower face of the filters.

#### 2.2.3. Phagocytosis

For this, the technique described by De la Fuente [16] was used. Aliquots of 200μL neutrophil suspension were incubated on migration inhibition factor (MIF) plates for 30 min. The adherent monolayer was washed with Hank’s solution at 37 °C, and 20μL of latex beads (1.09 μm diluted 1% PBS, Sigma-Aldrich, St. Louis, MO, USA) were added. After 30 min incubation, the sample was fixed with 50% methanol and stained with Giemsa (Sigma-Aldrich, St. Louis, MO, USA). The number of particles per 100 neutrophils (phagocytic index) and the percentage of neutrophils that ingested at least one particle (phagocytic efficiency) were determined by optical microscopy (100×).

#### 2.2.4. Natural Killer Activity

For the evaluation of NK activity, an enzymatic colorimetric kit (Cytotox 96 TM, Promega) based on the determination of lactate dehydrogenase (LDH) released by cytolysis of target cells using tetrazolium salts was performed. The suspension was added to 96-well U-bottom culture plates with target cells (human K-562 lymphoma cells) in a 10:1 ratio. After 4 h of incubation, LDH was measured by the addition of the enzyme substrate at an absorbance of 490 nm.

The formula to calculate this function is as follows:

$$Lysis\;\%=\frac{Problem\;lysis-Effector\;cells\;spontaneous\;lysis-Tumor\;cells\;spontaneous\;lysis}{Tumor\;cells\;total\;lysis-Tumor\;cells\;spontaneous\;lysis}\times 100$$

The results were expressed as the percentage of tumor cells killed (% lysis), as previously described [16].

#### 2.2.5. Lymphoproliferation

Lymphocyte proliferation both under basal conditions and in response to the mitogens Phytohemagglutinin (PHA) and Lipopolysaccharide (LPS) was assessed using a commercial kit: cell proliferation ELISA, BrdU (colorimetric) (Roche Applied Science). This kit assesses the incorporation of 5-bromo-2-deoxyuridine (BrdU), a thymidine analog, into the DNA of proliferating lymphocytes. For this purpose, 200 μL/well of lymphocyte suspensions adjusted to 10^6^ lymphocytes/mL of RPMI supplemented with gentamicin (1 mg/mL) and 10% fetal bovine serum (Gibco) previously decomplementarized by heating for 30 min at 56 °C were added to sterile 96-well plates. To the wells, 20 μL of RPMI complete medium was added for the basal condition and 20 μL of PHA or LPS (1 μg/mL) to assess the response to these mitogens. After 48 h of incubation, 100 μL of each well was collected for cytokine measurement. The volume was recovered with fresh medium, and BrdU was added. After that, the kit protocol was started to assess its incorporation into DNA. The results are expressed in absorbance units (AU). In addition, the percentage of stimulation was assessed, that is, mitogen-stimulated lymphoproliferation divided by basal lymphoproliferation × 100.

### 2.3. Evaluation of Redox Parameters

To evaluate the redox parameters (glutathione reductase and peroxidase activities, oxidized and reduced glutathione concentrations, and thiobarbituric acid reactive substance concentration), whole blood cells (including erythrocytes and total leukocytes) were used. For this, blood samples were centrifuged at 1300× *g* for 20 min. Then, plasma and whole blood cells were separated, and the pellets were reconstituted with RPMI+ medium and frozen at −80 °C until use [32].

#### 2.3.1. Glutathione Reductase Activity

Whole blood cells were resuspended in oxygen-free phosphate buffer (pH 7.4, 50 mM with 6.3 nM EDTA). Then, they were sonicated and centrifuged. Supernatants (1:5) were used for the reaction together with GSSG 80 mM as substrate, as previously described [32]. The oxidation of NADPH was measured at 340 nm for 4 min. The results were expressed as mU of glutathione reductase (GR)/mg protein.

#### 2.3.2. Glutathione Peroxidase Activity

Whole blood cells were resuspended in oxygen-free phosphate buffer (pH 7.4 50 mM). Then, they were sonicated and centrifuged. Supernatants (1:30) were used for the enzymatic reaction together with cumene hydroperoxide as a substrate, as previously described [32]. Oxidation of NADPH was measured at 340 nm for 5 min. The results were expressed as mU of glutathione peroxidase (GPx)/mg protein.

#### 2.3.3. Oxidized (GSSG) and Reduced (GSH) Glutathione Concentrations

Whole blood cells were resuspended in phosphate buffer (pH 8, 50 mM EDTA 0.1 M). Then, they were sonicated and centrifuged. Supernatants were used for the quantification of both oxidized (GSSG) and reduced (GSH) glutathione by the reaction capacity that they have with o-phthalaldehyde at pH 12 and pH 8, respectively, resulting in a fluorescent compound measured at 420 nm, as previously described [32]. Results were expressed as nmol of GSSG and GSH/mg protein. Moreover, the GSSG/GSH ratio was calculated.

#### 2.3.4. Concentration of Thiobarbituric Acid Reactive Substances (TBARs)

Quantification of TBARs was performed using the commercial kit: Lipid Peroxidation Assay Kit (Biovision, San Francisco, CA, USA). Whole blood cells were resuspended in lysis buffer (containing BHT 0.1 mM), sonicated, and centrifuged. Supernatants were mixed with thiobarbituric acid (TBA) and incubated in a water bath at 95 °C for 60 min. Then, samples were centrifuged, supernatants collected, and absorbance was measured at 532 nm, as previously described [32]. Results were expressed as nmol TBARs/mg protein.

#### 2.3.5. Protein Quantification

The protein content of each sample was evaluated to report all oxidative stress parameters in mg of protein. For this, protein assessment was carried out on the same supernatants collected from the analysis of the different redox parameters. Protein quantification was performed by the bicinchoninic acid (BCA) method, using the BCA kit, which is based on the reduction of Cu^2+^, generating Cu^+^ ions that bind to BCA and form a colored compound that absorbs light at 562 nm. The results were expressed in mg protein/mL [32].

### 2.4. Biological Age Determination

To estimate the biological age of each participant, the Immunity Clock model [16] and the redox signature were applied [32,33], which included some of the immune function and redox parameters evaluated in this study. The Immunity Clock formula is as follows: ImmunolAge = 93.943 − 0.230 × Natural Killer activity − 0.001 × lymphoproliferative response to PHA − 0.022 × neutrophil chemotaxis − 0.020 × phagocytic index − 0.019 × lymphocyte chemotaxis. The RedOx signature includes the following parameters: glutathione reductase and glutathione peroxidase activities, concentration of oxidized (GSSG) and reduced (GSH) glutathione, GSSG/GSH ratio and concentration of thiobarbituric acid reactive substances.

### 2.5. Cytokine Measurement

For cytokine measurement, plasma and lymphocyte culture samples in basal and stimulated conditions (PHA) were used. The concentration of TNF-α, IL-1β, IL-6, IL-10, and IL-2 were measured simultaneously in these samples by multiplex luminometry (Milliplex^®^ (Darmstadt, Germany) MAP Human High Sensitivity T Cell Magnetic Bead Panel—HSTCMAG-28SK, Millipore), according to the manufacturer’s instructions. Results were expressed as pg/mL.

### 2.6. Statistical Analysis

Statistical analysis was performed in GraphPad Prism 10.1.1. Data were represented as mean ± standard deviation (SD). The normality of the samples and homogeneity of the variances were checked using the Kolmogorov-Smirnov test and Levene test, respectively. Comparisons between the initial and post-treatment conditions were made by the dependent-samples t-test, and comparisons between sexes were made by the independent-samples t-test according to the compatibility of the data with normal distribution. *p* < 0.05 was considered statistically significant.

## 3. Results

**Figure 1:**
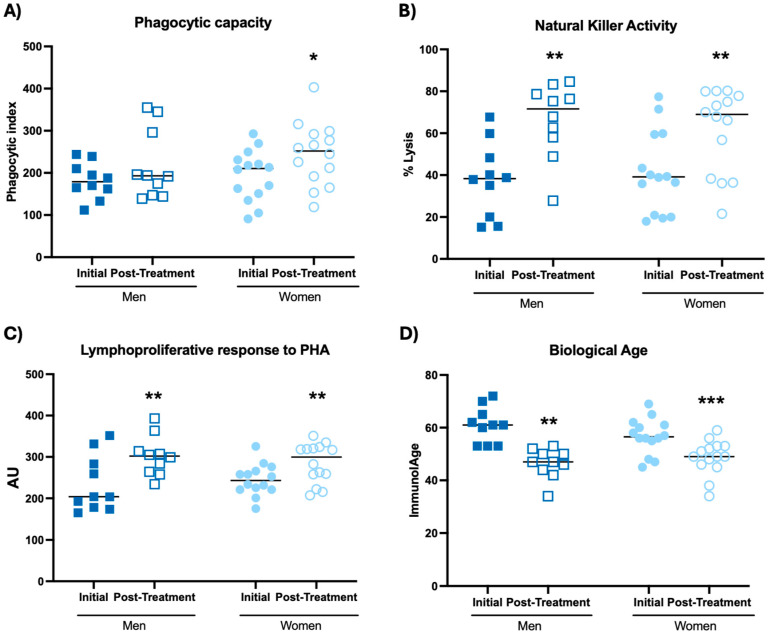
Immune function in peripheral blood leukocytes of participants before and after probiotic blend intakes. (**A**) Phagocytic capacity of neutrophils. (**B**) Natural killer activity. (**C**) Lymphoproliferative response to PHA. (**D**) Biological age. * *p* < 0.05, ** *p* < 0.01, *** *p* < 0.001 compared to the initial condition. PHA: Phytohemagglutinin; AU: Absorbance units.

The results related to immune function are shown in Figure 1 and Table 1.

It can be observed that, after taking the probiotic blend, both men and women increased their natural killer activity (Figure 1B, *p* < 0.01) and the proliferative response of lymphocytes in response to PHA (Figure 1C, *p* < 0.01) with respect to the initial time. Moreover, after intake of the probiotic blend, women also increased their phagocytic capacity (Figure 1A, *p* < 0.05; Table 1, *p* < 0.01), while men increased their basal lymphoproliferation (Table 1, *p* < 0.01) with respect to the initial time. However, ingestion of the probiotic blend did not result in increased chemotactic capacity, either of neutrophils or lymphocytes or in increased lymphoproliferation in response to LPS in either men or women (Table 1). Moreover, the potentiation generated by the probiotic blend in these immune functions implies that both men and women decreased their biological age by an average of 11 years (11 ± 6) (Figure 1D, *p* < 0.01; *p* < 0.001, respectively) with respect to the initial time. It is important to mention that at the initial time, the participants showed a higher biological age (58 ± 7) than their chronological age (48 ± 8) (Figure 1D).

**Figure 2:**
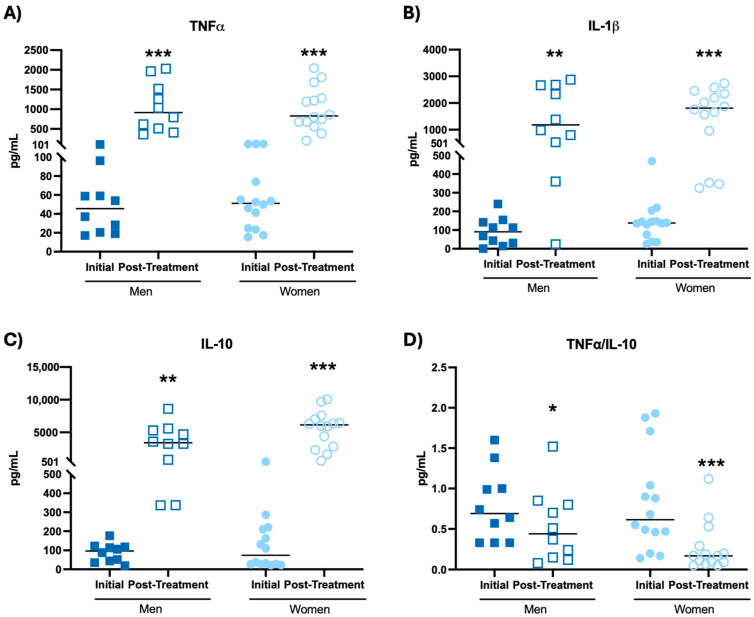
Cytokine concentrations (pg/mL) in mononuclear cell culture supernatants at the basal condition of participants before and after probiotic blend intakes. (**A**) TNF-α concentration. (**B**) IL-1β concentration. (**C**) IL-10 concentration. (**D**) TNF-α/IL-10 ratio. * *p* < 0.05, ** *p* < 0.01, *** *p* < 0.001 compared to the initial condition.

The results obtained from the assessment of cytokine concentration in mononuclear cell cultures in basal condition are shown in Figure 2 and Table 1. 

In both men and women, an increase in TNF-α (Figure 2A, *p* < 0.001), IL-1β (Figure 2B, *p* < 0.01; *p* < 0.001, respectively), IL-10 (Figure 2C, *p* < 0.01; *p* < 0.001, respectively), IL-6 (Table 1, *p* < 0.05; *p* < 0.01, respectively) and IL-2 (Table 1, *p* < 0.001) concentrations, as well as a decrease in the TNF-α/IL-10 ratio (Figure 2D, *p* < 0.05; *p* < 0.001, respectively) after the probiotic blend intake were observed with respect to the initial time.

**Figure 3:**
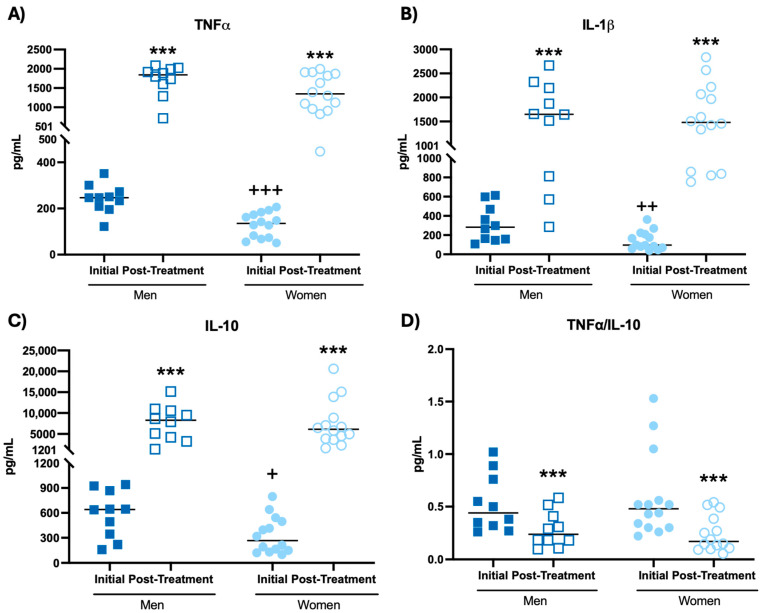
Cytokine concentrations (pg/mL) in mononuclear cell culture supernatants at the Phytohemagglutinin-stimulated condition of participants before and after probiotic blend intakes. (**A**) TNF-α concentration. (**B**) IL-1β concentration. (**C**) IL-10 concentration. (**D**) TNF-α/IL-10 ratio. *** *p* < 0.001 compared to the initial condition. + *p* < 0.05, ++ *p* < 0.01, +++ *p* < 0.001 compared to men at the same condition.

Moreover, the results obtained from the assessment of cytokine concentrations in mononuclear cell cultures after Phytohemagglutinin (PHA) stimulation are shown in Figure 3 and Table 1. 

The results show that after intake of the probiotic blend, both men and women increased the concentrations of TNF-α (Figure 3A, *p* < 0.001), IL-1β (Figure 3B, *p* < 0.01; *p* < 0.001, respectively), IL-10 (Figure 3C, *p* < 0.01; *p* < 0.001, respectively) and IL-2 (Table 1, *p* < 0.001), together with a decrease in the TNF-α/IL-10 ratio (Figure 3D, *p* < 0.001) compared to the initial time. Women also increased the IL-6 concentration (Table 1, *p* < 0.001) after probiotic blend intake with respect to the initial time. It is worth mentioning that women at the initial time showed a lower concentration of TNF-α (Figure 3A, *p* < 0.001), IL-1β (Figure 3B, *p* < 0.01), IL-10 (Figure 3C, *p* < 0.05) and IL-6 (Table 1, *p* < 0.001) in mononuclear cell cultures after stimulation with PHA, compared to men at their initial time.

**Figure 4:**
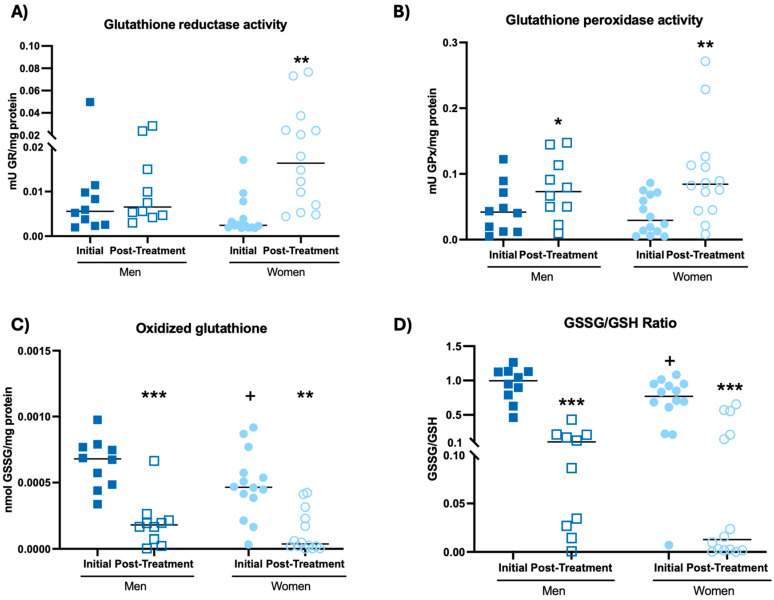
Redox parameters in whole blood cells of participants before and after probiotic blend intakes. (**A**) Glutathione reductase activity. (**B**) Glutathione peroxidase activity. (**C**) Oxidized glutathione concentration. (**D**) GSSG/GSH ratio. GSH: reduced glutathione. * *p* < 0.05, ** *p* < 0.01, *** *p* < 0.001 compared to the initial condition. + *p* < 0.05 compared to men in the same condition. GR: glutathione reductase activity; GPx: glutathione peroxidase activity; GSSG: oxidized glutathione.

The results obtained from the redox state assessment are shown in Figure 4 and Table 2.

The intake of the probiotic blend allowed men and women to increase the enzymatic activity of glutathione peroxidase (Figure 4B, *p* < 0.05; *p* < 0.01, respectively) and the concentration of reduced glutathione (GSH) (Table 2, *p* < 0.05; 0.01, respectively), as well as decrease oxidized glutathione (GSSG) concentration (Figure 4C, *p* < 0.001; *p*<0.01, respectively) and GSSG/GSH ratio (Figure 4D, *p* < 0.001) with respect to the initial time. In addition, women increased glutathione reductase activity after probiotic blend intake (Figure 4A, *p* < 0.01) with respect to initial time. The probiotic blend intake did not affect lipid peroxidation (Table 2). Furthermore, women at the initial time showed a lower GSSG concentration (Figure 4C, *p* < 0.05), as well as a lower GSSG/GSH ratio (Figure 4D, *p* < 0.05) with respect to men at their initial time. Finally, it could be observed that the effects on the redox parameters after taking the probiotic blend implied that both men and women decreased their redox signatures (Table 2, *p* < 0.001).

**Figure 5:**
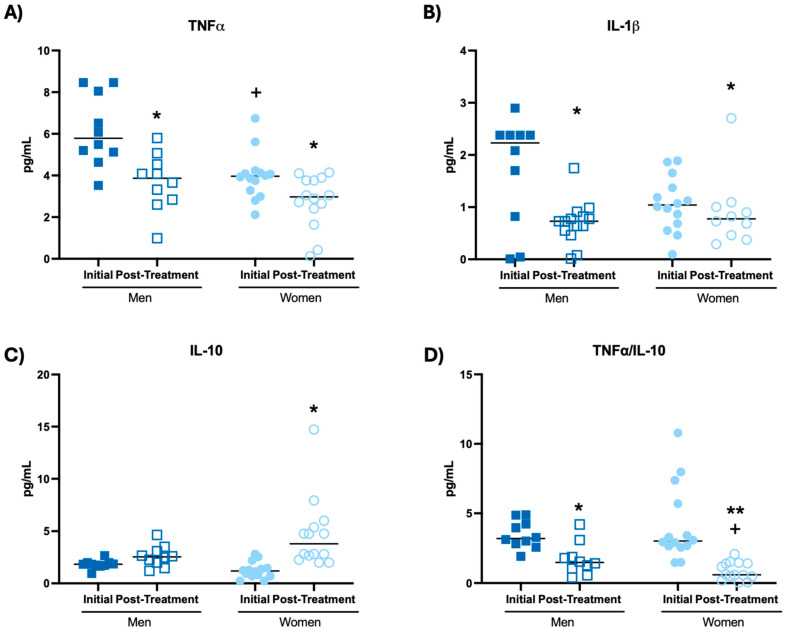
Cytokine concentrations (pg/mL) in plasma of participants before and after probiotic blend intakes. (**A**) TNF-α concentration. (**B**) IL-1β concentration. (**C**) IL-10 concentration. (**D**) TNF-α/IL-10 ratio. * *p* < 0.05, ** *p* < 0.01 compared to the initial condition. + *p* < 0.05 compared to men in the same condition.

Finally, the results obtained from the evaluation of cytokine concentration in plasma are shown in Figure 5 and Table 2.

It can be observed that in plasma, after the intake of the probiotic blend, both men and women decreased the concentration of TNF-α (Figure 5A, *p* < 0.05), IL-1β (Figure 5B, *p* < 0.05) and the TNF-α/IL-10 ratio (Figure 5D, *p* < 0.05; *p* < 0.01, respectively) and increased the concentration of IL-6 (Table 2, *p* < 0.05) with respect to the initial time. Furthermore, women also increased the IL-10 concentration (Figure 5C, *p* < 0.05) and decreased the concentration of IL-2 (Table 2, *p* < 0.05) after probiotic blend intake with respect to the initial time. In addition, women showed lower TNF-α (Figure 5A, *p* < 0.05) and higher IL-2 concentrations at the initial time, as well as a lower TNF-α/IL-10 ratio (Figure 5D, *p* < 0.05) after probiotic intake with respect to men in the same condition.

## 4. Discussion

This study is the first to demonstrate the effect that daily consumption of a probiotic blend for 2 months can have not only on the immunity of the participants, but also on the oxidative state, inflammatory profile, and rate of aging.

The results show that the intake of this compound composed of three probiotic strains: *Bifidobacterium animalis* subsp. *lactis* BSO1, *Lactobacillus reuteri* LRE02, *Lactobacillus plantarum* LP14, and vitamin D3 improves the immune function of both men and women. Thus, although it has no effect on the chemotactic capacity of neutrophils and lymphocytes, it increases the phagocytic capacity of female neutrophils and increases the natural killer activity and lymphoproliferative capacity of lymphocytes in response to PHA in both men and women. These results agree with previous studies where it has been observed that the consumption of *Bifidobacterium lactis* improves the phagocytic capacity of the cells [25,34]. In addition, it has also been previously described that the intake of *Bifidobacterium animalis* ssp. *Lactis* and *Lactobacillus plantarum*, both individually and together, also stimulates natural killer activity [34,35,36]. Regarding parameters more related to adaptive immunity, we found that consumption of the probiotic blend stimulates the lymphoproliferative response in the presence of the mitogen PHA, a function carried out mainly by T lymphocytes [37]. Based on this, it has been observed that the strain of the species *Lactobacillus plantarum* could be responsible for enhancing this function [38]. Furthermore, it can be observed that there is an increase in basal lymphoproliferation in men after taking the probiotic blend. This could also be due to the presence of *Lactobacillus plantarum* and *Bifidobacterium lactis*, as the intake of these probiotic strains has been described to stimulate the proliferation of mononuclear cells in culture, especially enhancing the expression of CD4(+) and CD8(+) T lymphocytes [39,40]. However, no improvement in proliferation in response to LPS, mediated mainly by B lymphocytes, was observed, as is the case with supplementation with other probiotic strains, such as *Lactobacillus rhamnosus* GG or *Akkermansia mucciniphila* [19,41].

The improvement of all these immune functions, which are included in the prediction model of biological age and Immunity Clock [16], could explain that the intake of this probiotic blend slows down the rate of aging of the participants. It was observed that the participants before the intake of the compound had a biological age higher than their chronological age, which could be due, among other things, to poor lifestyle habits or poor stress regulation. As these probiotics improved the functions of the studied immune cells, which are markers of biological age and modulate the rate of aging [17], the participants managed to slow down this process by reducing their biological age by an average of 10 years.

Furthermore, the intake of this probiotic blend also showed effects on cytokine release in mononuclear cell cultures. Thus, both in cultures under basal conditions and in those stimulated with PHA, an increase in pro-inflammatory TNF-α, IL-1β, and IL-6, and anti-inflammatory IL-10 and regulatory IL-2 cytokines [42,43,44] could be observed. However, despite all of these increases, participants reduced their inflammation, as shown by the TNF-α/IL-10 ratio, which has been proposed as a good marker of the degree of inflammatory stress in an individual [45]. It is noteworthy that, although the release of these cytokines increased in both conditions, the release was higher in response to PHA, as would be expected since it stimulates lymphocyte proliferation and, therefore, the release of cytokines among other metabolites [37]. Although it is true that under basal conditions, it would be expected that there would not be a significant increase in the release of these cytokines, it has been described that the intake of certain probiotic strains, such as *Bifidobacterium animalis*, *Lactobacillus plantarum*, and *Lactobacillus reuteri*, is capable of stimulating the release of cytokines favoring an anti-inflammatory profile [26,36,40,46,47,48,49].

Moreover, the overall inflammatory profile of the participants was studied in plasma, and a decrease in the pro-inflammatory cytokines TNF-α and IL-1β and an increase in the anti-inflammatory IL-10 was observed after the probiotic intake, thus decreasing the TNF-α/IL-10 ratio. Therefore, as happens in cell cultures, these probiotic strains exerted an anti-inflammatory effect [30,31,48,50]. Curiously, an increase in IL-6 was observed in plasma after taking the probiotic blend. This could be due to the fact that the body would be favoring the differentiation of B lymphocytes through the production of this cytokine in response to the presence of these probiotic strains [51].

In addition, it could be observed how the intake of this probiotic blend improved the redox state of the participants, decreasing the concentration of oxidative compounds and increasing the presence and activity of antioxidant compounds. Other studies have shown that *Bifidobacterium animalis*, *Lactobacillus plantarum*, and *Lactobacillus reuteri* are able to regulate the oxidative state of the organism by increasing the activities of glutathione reductase, glutathione peroxidase, superoxide dismutase, and catalase, and the concentration of reduced glutathione, as well as decreasing oxidative compounds such as oxidized glutathione, nitric oxide, malondialdehyde, and lipid peroxidation [30,31,52,53,54,55,56]. In our study, similar effects were observed except for the decrease in lipid peroxidation, in which we found no significant differences. Moreover, the effects observed on the components of the glutathione cycle would also be beneficial for promoting the maintenance and colonization of the probiotic strains in the organism, since it has been observed that certain probiotic strains, such as *Lactobacillus reuteri*, need glutathione as a nutrient for their survival [57]. Finally, when the redox signature of the participants was studied, it was observed that after the consumption of probiotics, it also decreased, indicating that the rate of aging was decreasing [32,33].

It is important to note that although the contribution of the three probiotic strains to the observed effects was evident, the compound also contains vitamin D3, which is capable of stimulating the immune system and has antioxidant and anti-inflammatory properties [58,59], thus contributing to the potentiation of the effects produced by the probiotics.

Finally, it is worth mentioning that in the present study, differences in the positive effects of probiotics were found according to sex, as the effects were more noticeable in women. The differences between men and women have been extensively studied in terms of microbiota and response to probiotics [60,61,62,63], as well as in the functioning of the homeostatic systems and the oxidative and inflammatory profile, which has been related to the higher life expectancy of women compared to men [64]. Therefore, an increase in the duration of probiotic intake in men could be suggested to achieve positive effects as significant as those observed in women.

However, although the results of the present study highlight the potential of these probiotic strains together with vitamin D3 to improve immunity, reduce the oxidative and inflammatory profile, and slow down the rate of aging, it should not be forgotten that this is a pilot study. Therefore, it would be convenient to carry out the study again with a larger sample size, as well as an increase in the duration of probiotic intake for men. It would also be interesting to assess the role of vitamin D3 in the results obtained.

## Figures and Tables

**Table 1:** Immune function in peripheral blood leukocytes and cytokine release in monocyte cultures at basal and Phytohemagglutinin-stimulated conditions of participants before and after probiotic blend intakes.

	Men		Women	
	Initial	Post-Treatment	Initial	Post-Treatment
Neutrophile functions				
Chemotaxis index (C.I)	471 ± 252	549 ± 232	453 ± 215	518 ± 265
Phagocytic efficacy	67 ± 11	70 ± 4	66 ± 8	71 ± 6 **
Lymphocyte functions				
Chemotaxis index (C.I)	433 ± 240	513 ± 177	482 ± 181	448 ± 181
Lymphoproliferation				
Basal proliferative response (AU)	203 ± 23	240 ± 33 **	201 ± 19	218 ± 47
Proliferative response to LPS (AU)	208 ± 44	248 ± 61	201 ± 28	217 ± 36
% Stimulation with PHA	115 ± 28	127 ± 21	122 ± 16	134 ± 27
% Stimulation with LPS	102 ± 16	105 ± 27	100 ± 14	101 ± 16
Cytokine concentration in mononuclear cell cultures				
Basal condition				
IL-6 (pg/mL)	1037 ± 701	1744 ± 449 *	900 ± 695	1712 ± 947 **
IL-2 (pg/mL)	0.72 ± 0.4	241 ± 146 ***	1.18 ± 1	325 ± 176 ***
PHA-stimulated condition				
IL-6 (pg/mL)	1585 ± 363	1557 ± 147	1059 ± 361 +++	1583 ± 127 ***
IL-2 (pg/mL)	2.1 ± 1.4	374 ± 268 ***	1.1 ± 0.7	644 ± 452 ***

Each value represents the mean ± standard deviation. * *p* < 0.05, ** *p* < 0.01, *** *p* < 0.001 compared to the initial condition. +++ *p* < 0.001 compared to men in the same condition. PHA: Phytohemagglutinin; LPS: Lipopolysaccharide. AU: Absorbance units.

**Table 2:** Redox parameters in whole blood cells and cytokine concentrations in plasma of participants before and after probiotic blend intakes.

	Men		Women	
	Initial	Post-Treatment	Initial	Post-Treatment
Antioxidant compounds				
Reduced glutathione concentration (nmol GSH/mg protein)	0.0007 ± 0.0001	0.003 ± 0.002 *	0.001 ± 0.0009	0.005 ± 0.003 **
Oxidant compounds				
TBAR concentration (nmol TBARs/mg protein)	0.06 ± 0.2	0.06 ± 0.02	0.05 ± 0.02	0.04 ± 0.02
RedOx signature (years)	55 ± 0.8	51 ± 0.3 ***	54 ± 0.6	50 ± 0.3 ***
Cytokine concentration (plasma)				
IL-6 (pg/mL)	0.4 ± 0.2	3.3 ± 2.5 *	1.1 ± 0.9	2.8 ± 2.2 *
IL-2 (pg/mL)	1.4 ± 0.6	1.3 ± 0.7	3.1 ± 0.7 +	1.5 ± 0.9 *

Each value represents the mean ± standard deviation. * *p* < 0.05, ** *p* < 0.01, *** *p* < 0.001 compared to the initial condition. + *p* < 0.05 compared to men in the same condition. GSH: reduced glutathione; TBARs: thiobarbituric acid reactive substances.

## Data Availability

Data will be available upon request to Judith Félix (jufelix@ucm.es).

## References

[1] De la Fuente M. (2021). The Role of the Microbiota-Gut-Brain Axis in the Health and Illness Condition: A Focus on Alzheimer’s Disease. J. Alzheimers Dis..

[2] Roy R., Singh S.K. (2024). The Microbiome Modulates the Immune System to Influence Cancer Therapy. Cancers.

[3] Mirmohammadali S.N., Gallant K.M.H., Biruete A. (2024). Oh, My Gut! New insights on the role of the gastrointestinal tract and the gut microbiome in chronic kidney disease-mineral and bone disorder. Curr. Opin. Nephrol. Hypertens..

[4] Vidal-Gallardo A., Méndez Benítez J.E., Flores Rios L., Ochoa Meza L.F., Mata Pérez R.A., Martínez Romero E., Vargas Beltran A.M., Beltran Hernandez J.L., Banegas D., Perez B. (2024). The Role of Gut Microbiome in the Pathogenesis and the Treatment of Inflammatory Bowel Diseases. Cureus.

[5] Sánchez-Pellicer P., Eguren-Michelena C., García-Gavín J., Llamas-Velasco M., Navarro-Moratalla L., Núñez-Delegido E., Agüera-Santos J., Navarro-López V. (2023). Rosacea. Microbiome and probiotics: The gut-skin axis. Front. Microbiol..

[6] MacKay M., Yang B.H., Dursun S.M., Baker G.B. (2024). The Gut-Brain Axis and the Microbiome in Anxiety Disorders, Post-Traumatic Stress Disorder and Obsessive-Compulsive Disorder. Curr. Neuropharmacol..

[7] De la Fuente M., Miquel J. (2009). An update of the oxidation-inflammation theory of aging: The involvement of the immune system in oxi-inflamm-aging. Curr. Pharm. Des..

[8] Claesson M.J., Jeffery I.B., Conde S., Power S.E., O’Connor E.M., Cusack S., Harris H.M., Coakley M., Lakshminarayanan B., O’Sullivan O. (2012). Gut microbiota composition correlates with diet and health in the elderly. Nature.

[9] Candela M., Biagi E., Brigidi P., O’Toole P.W., De Vos W.M. (2014). Maintenance of a healthy trajectory of the intestinal microbiome during aging: A dietary approach. Mech. Ageing Dev..

[10] Clements S.J.R., Carding S. (2018). Diet, the intestinal microbiota, and immune health in aging. Crit. Rev. Food Sci. Nutr..

[11] Salazar N., Valdés-Varela L., González S., Gueimonde M., de Los Reyes-Gavilán C.G. (2017). Nutrition and the gut microbiome in the elderly. Gut Microbes.

[12] Waitzberg D., Guarner F., Hojsak I., Ianiro G., Polk D.B., Sokol H. (2024). Can the Evidence-Based Use of Probiotics (Notably Saccharomyces boulardii CNCM I-745 and Lactobacillus rhamnosus GG) Mitigate the Clinical Effects of Antibiotic-Associated Dysbiosis?. Adv. Ther..

[13] Joldrichsen M.R., Kim E., Steiner H.E., Jeong Y.J., Premanandan C., Hsueh W., Ziouzenkova O., Cormet-Boyaka E., Boyaka P.N. (2024). Loss of Paneth cells dysregulates gut ILC subsets and enhances weight gain response to high fat diet in a mouse model. bioRxiv.

[14] Hunsche C., Hernandez O., De la Fuente M. (2016). Impaired Immune Response in Old Mice Suffering from Obesity and Premature Immunosenescence in Adulthood. J. Gerontol A-Biol..

[15] Martínez de Toda I., Ceprián N., Díaz-Del Cerro E., De la Fuente M. (2021). The Role of Immune Cells in Oxi-Inflamm-Aging. Cells.

[16] Martínez de Toda I., Vida C., Díaz-Del Cerro E., De la Fuente M. (2021). The Immunity Clock. J. Gerontol A-Biol..

[17] Martínez de Toda I., Maté I., Vida C., Cruces J., De la Fuente M. (2016). Immune function parameters as markers of biological age and predictors of longevity. Aging.

[18] Félix J., Martínez de Toda I., Díaz-Del Cerro E., Gil-Agudo F., De la Fuente M. (2024). The immunity and redox clocks in mice, markers of lifespan. Sci. Rep..

[19] Díaz-Del Cerro E.D., Lambea M., Félix J., Salazar N., Gueimonde M., De la Fuente M. (2022). Daily ingestion of Akkermansia mucciniphila for one month promotes healthy aging and increases lifespan in old female mice. Biogerontology.

[20] Hunsche C., Cruces J., De la Fuente M. (2019). Improvement of Redox State and Functions of Immune Cells as Well as of Behavioral Response in Aged Mice After Two-Week Supplementation of Fermented Milk with Probiotics. Curr. Microbiol..

[21] Penha Rodrigues Pereira E., Silva da Graça J., Manfrinato Ferreira B., Fasura Balthazar C., Xavier-Santos D., França Bezerril F., Magnani M., Sant’Ana A.S. (2024). What are the main obstacles to turning foods healthier through probiotics incorporation? a review of functionalization of foods by probiotics and bioactive metabolites. Food Res. Int..

[22] Zhou P., Chen C., Patil S., Dong S. (2024). Unveiling the therapeutic symphony of probiotics, prebiotics, and postbiotics in gut-immune harmony. Front. Nutr..

[23] Li L., Li M., Chen Y., Yu Z., Cheng P., Yu Z., Cheng W., Zhang W., Wang Z., Gao X. (2024). Function and therapeutic prospects of next-generation probiotic Akkermansia muciniphila in infectious diseases. Front. Microbiol..

[24] Arboleya S., Watkins C., Stanton C., Ross R.P. (2016). Gut Bifidobacteria Populations in Human Health and Aging. Front. Microbiol..

[25] Miller L.E., Lehtoranta L., Lehtinen M.J. (2017). The Effect of Bifidobacterium animalis ssp. lactis HN019 on Cellular Immune Function in Healthy Elderly Subjects: Systematic Review and Meta-Analysis. Nutrients.

[26] Zhao W., Peng C., Sakandar H.A., Kwok L.Y., Zhang W. (2021). Meta-Analysis: Randomized Trials of Lactobacillus plantarum on Immune Regulation Over the Last Decades. Front. Immunol..

[27] Vijayan S., Kandi V., Palacholla P.S., Rajendran R., Jarugu C., Ca J., Pravallika M., Reddy S.C., Sucharitha A.S. (2024). Probiotics in Allergy and Immunological Diseases: A Comprehensive Review. Cureus.

[28] Clancy R. (2003). Immunobiotics and the probiotic evolution. FEMS Immunol. Med. Microbiol..

[29] Tsai Y.C., Cheng L.H., Liu Y.W., Jeng O.J., Lee Y.K. (2021). Gerobiotics: Probiotics targeting fundamental aging processes. Biosci. Microbiota Food Health.

[30] Tang Q., Yi H., Hong W., Wu Q., Yang X., Hu S., Xiong Y., Wang L., Jiang Z. (2021). Comparative Effects of *L. plantarum* CGMCC 1258 and *L. reuteri* LR1 on Growth Performance, Antioxidant Function, and Intestinal Immunity in Weaned Pigs. Front. Vet. Sci..

[31] Tang J., Guo C., Gong F. (2019). Protective effect of Lactobacillus reuteri against oxidative stress in neonatal mice with necrotizing enterocolitis. Nan Fang Yi Ke Da Xue Xue Bao.

[32] Diaz-Del Cerro E., Martinez de Toda I., Félix J., Baca A., De la Fuente M. (2023). Components of the Glutathione Cycle as Markers of Biological Age: An Approach to Clinical Application in Aging. Antioxidants.

[33] Martínez de Toda I., Vida C., Garrido A., De la Fuente M. (2020). Redox Parameters as Markers of the Rate of Aging and Predictors of Life Span. J. Gerontol A-Biol..

[34] Moon P.D., Lee J.S., Kim H.Y., Han N.R., Kang I., Kim H.M., Jeong H.J. (2019). Heat-treated Lactobacillus plantarum increases the immune responses through activation of natural killer cells and macrophages on in vivo and in vitro models. J. Med. Microbiol..

[35] Lee A., Lee Y.J., Yoo H.J., Kim M., Chang Y., Lee D.S., Lee J.H. (2017). Consumption of Dairy Yogurt Containing *Lactobacillus paracasei* ssp. paracasei, *Bifidobacterium animalis* ssp. lactis and Heat-Treated Lactobacillus plantarum Improves Immune Function Including Natural Killer Cell Activity. Nutrients.

[36] Kim J.Y., Kim J.Y., Kim H., Moon E.C., Heo K., Shim J.J., Lee J.L. (2022). Immunostimulatory effects of dairy probiotic strains Bifidobacterium animalis ssp. lactis HY8002 and Lactobacillus plantarum HY7717. J. Anim. Sci. Technol..

[37] Movafagh A., Heydary H., Mortazavi-Tabatabaei S.A., Azargashb E. (2011). The Significance Application of Indigenous Phytohemagglutinin (PHA) Mitogen on Metaphase and Cell Culture Procedure. Iran. J. Pharm. Res..

[38] Hirose Y., Murosaki S., Yamamoto Y., Yoshikai Y., Tsuru T. (2006). Daily intake of heat-killed Lactobacillus plantarum L-137 augments acquired immunity in healthy adults. J. Nutr..

[39] Arunachalam K., Gill H.S., Chandra R.K. (2000). Enhancement of natural immune function by dietary consumption of Bifidobacterium lactis (HN019). Eur. J. Clin. Nutr..

[40] Liu J., Gao K., Li D., Zeng Y., Chen X., Liang X., Fang C., Gu Y., Wang C., Yang Y. (2022). Recombinant invasive Lactobacillus plantarum expressing the J subgroup avian leukosis virus Gp85 protein induces protection against avian leukosis in chickens. Appl. Microbiol. Biotechnol..

[41] Bornholdt J., Broholm C., Chen Y., Rago A., Sloth S., Hendel J., Melsæther C., Müller C.V., Juul Nielsen M., Strickertsson J. (2020). Personalized B cell response to the Lactobacillus rhamnosus GG probiotic in healthy human subjects: A randomized trial. Gut Microbes.

[42] Banchereau J., Pascual V., O’Garra A. (2012). From IL-2 to IL-37: The expanding spectrum of anti-inflammatory cytokines. Nat. Immunol..

[43] Al-Qahtani A.A., Alhamlan F.S., Al-Qahtani A.A. (2024). Pro-Inflammatory and Anti-Inflammatory Interleukins in Infectious Diseases: A Comprehensive Review. Trop. Med. Infect. Dis..

[44] Garrido A., de la Fuente M. (2022). Social environment improves the cytokine profile and lymphoproliferative response in chronologically old and prematurely aging mice. Mech. Ageing Dev..

[45] Martínez de Toda I., Vida C., De la Fuente M. (2017). An Appropriate Modulation of Lymphoproliferative Response and Cytokine Release as Possible Contributors to Longevity. Int. J. Mol. Sci..

[46] Wang Y., Xie Q., Zhang Y., Ma W., Ning K., Xiang J.Y., Cui J., Xiang H. (2020). Combination of probiotics with different functions alleviate DSS-induced colitis by regulating intestinal microbiota, IL-10, and barrier function. Appl. Microbiol. Biotechnol..

[47] DeMuri G.P., Lehtoranta L.M., Eickhoff J.C., Lehtinen M.J., Wald E.R. (2021). Ex vivo peripheral blood mononuclear cell response to R848 in children after supplementation with the probiotic Lactobacillus acidophilus NCFM/Bifidobacterium lactis Bi-07. Benef Microbes.

[48] Müller L., Kuhn T., Koch M., Fuhrmann G. (2021). Stimulation of Probiotic Bacteria Induces Release of Membrane Vesicles with Augmented Anti-inflammatory Activity. ACS Appl. Bio. Mater..

[49] Ferreira A.F., Braga R.L.L., Andrade M.F., Rosa A.C.P., Pereira-Manfro W.F. (2021). Synergistic immunomodulatory activity of probiotics Bifidobacterium animalis and Lactobacillus casei in enteroaggregative Escherichia coli (EAEC)-infected CACO-2 cells. Arq. Gastroenterol..

[50] Min Z., Xiaona H., Aziz T., Jian Z., Zhennai Y. (2020). Exopolysaccharides from Lactobacillus plantarum YW11 improve immune response and ameliorate inflammatory bowel disease symptoms. Acta Biochim. Pol..

[51] Hirano T. (1991). Interleukin 6 (IL-6) and its receptor: Their role in plasma cell neoplasias. Int. J. Cell Cloning.

[52] Vitheejongjaroen P., Kasorn A., Puttarat N., Loison F., Taweechotipatr M. (2022). Bifidobacterium animalis MSMC83 Improves Oxidative Stress and Gut Microbiota in D-Galactose-Induced Rats. Antioxidants.

[53] Izuddin W.I., Humam A.M., Loh T.C., Foo H.L., Samsudin A.A. (2020). Dietary Postbiotic Lactobacillus plantarum Improves Serum and Ruminal Antioxidant Activity and Upregulates Hepatic Antioxidant Enzymes and Ruminal Barrier Function in Post-Weaning Lambs. Antioxidants.

[54] Ahn S.I., Cho S., Choi N.J. (2020). Effect of dietary probiotics on colon length in an inflammatory bowel disease-induced murine model: A meta-analysis. J. Dairy Sci..

[55] Xu R., Shang N., Li P. (2011). In vitro and in vivo antioxidant activity of exopolysaccharide fractions from Bifidobacterium animalis RH. Anaerobe.

[56] Li F., Zhang B., Zhang Y., Zhang X., Usman S., Ding Z., Hao L., Guo X. (2022). Probiotic effect of ferulic acid esterase-producing Lactobacillus plantarum inoculated alfalfa silage on digestion, antioxidant, and immunity status of lactating dairy goats. Anim. Nutr..

[57] Lee K., Kim H.J., Rho B.S., Kang S.K., Choi Y.J. (2011). Effect of glutathione on growth of the probiotic bacterium Lactobacillus reuteri. Biochemistry.

[58] Mastali V.P., Hoseini R., Azizi M. (2023). The effect of short-term vitamin D on the antioxidant capacity following exhaustive aerobic exercise. Afr. Health Sci..

[59] Valdés-López J.F., Velilla P., Urcuqui-Inchima S. (2022). Vitamin D modulates the expression of Toll-like receptors and pro-inflammatory cytokines without affecting Chikungunya virus replication, in monocytes and macrophages. Acta Trop..

[60] Vemuri R., Sylvia K.E., Klein S.L., Forster S.C., Plebanski M., Eri R., Flanagan K.L. (2019). The microgenderome revealed: Sex differences in bidirectional interactions between the microbiota, hormones, immunity and disease susceptibility. Semin. Immunopathol..

[61] Holingue C., Budavari A.C., Rodriguez K.M., Zisman C.R., Windheim G., Fallin M.D. (2020). Sex Differences in the Gut-Brain Axis: Implications for Mental Health. Curr. Psychiat. Rep..

[62] Pace F., Watnick P.I. (2021). The Interplay of Sex Steroids, the Immune Response, and the Intestinal Microbiota. Trends Microbiol..

[63] Snigdha S., Ha K., Tsai P., Dinan T.G., Bartos J.D., Shahid M. (2022). Probiotics: Potential novel therapeutics for microbiota-gut-brain axis dysfunction across gender and lifespan. Pharmacol. Ther..

[64] Suarez L.M., Diaz-Del Cerro E., Felix J., Gonzalez-Sanchez M., Ceprian N., Guerra-Perez N., Novelle M.G., Martinez de Toda I., De la Fuente M. (2023). Sex differences in neuroimmunoendocrine communication. Involvement on longevity. Mech. Ageing Dev..

